# Understanding the perceptions and experiences of Certified Registered Nurse Anaesthetists regarding handovers: a focus group study

**DOI:** 10.1002/nop2.9

**Published:** 2014-11-15

**Authors:** Denise Testa, Susan Emery

**Affiliations:** ^1^ Boston College Nurse Anesthesia ProgramBostonMassachusetts02467

**Keywords:** Communication, handoff, handover, nurses, nursing, transfer of care

## Abstract

**Aim:**

The aim of this exploratory study was to gain further insight into the perceptions and experiences of Certified Registered Nurse Anaesthetists regarding intraoperative handovers of care.

**Background:**

Handovers of care often result in adverse events in hospitalized patients and this risk is increased in the operating room setting where handovers occur frequently. Handovers between nurse anaesthetists, who provide the majority of anaesthesia in the United States today, is under‐researched.

**Design:**

Focus groups with Certified Registered Nurse Anaesthetists.

**Methods:**

Two groups of nurse anaesthetists were recruited to participate in focus groups exploring their perception and experiences with intraoperative handovers of care. Content analysis was used to construe meaning from the context of the interviews. The findings were interpreted and discussed in a framework of Relationship‐Based Care.

**Findings:**

There were four main themes that emerged from the data: (1) characteristics of the setting are a threat to handover quality; (2) individual provider characteristics have an impact on handover quality; (3) The timing of the handover represents a threat to handover quality and (4) individual patient characteristics have an impact on handover quality.

**Conclusion:**

The specific threats to safe handover of care between nurse anaesthetists were perceived to fall into four major themes; this provides information needed to strengthen the environment of care and to improve safety in handover of care in the operating suite.

## Introduction

The exchange of information and continued responsibility for transferring patient care from one healthcare provider to another are essential component of communication in the healthcare delivery system. Synonymous terms for this exchange of information and transfer of responsibility include handover, handoff and transfer of care. Handovers between healthcare providers ideally result in the delivery of accurate and complete transfer of information about the patient and the care experience. It is estimated that 80% of serious medical errors involve miscommunication between caregivers when patient care is handed over (Beach 2008). Ineffective handovers contribute to increased threats to patient safety including medication errors, wrong site surgery and patient death (Sanchez 1997, Kluger & Bullock 2002). Certified Registered Nurse Anaesthetists (CRNAs) provide the majority of anaesthesia care in the United States and frequently hand over the care of patients to a colleague in the operating room. CRNAs are advanced practice nurses who are certified to practice the nursing specialty of anaesthesiology in all 50 states of the USA (Foster & Faut‐Callahan 2001). The practice of nurse anaesthesia is also well defined and implemented in Canada. The main role and scope of practice of CRNAs involves performing a pre‐anaesthetic assessment, developing and implementing an anaesthetic plan and facilitating emergence and recovery from anaesthesia. When care is handed over in the operating room, information regarding the patient's medical history, physical status such as vital signs, anaesthetic plan, surgical progress and potential complications is transferred from the outgoing to the incoming anaesthetist. The current practice involves handing over the care of the patient between anaesthetists for meal breaks and for end‐of‐shift relief and line‐by‐line coding. Thus, handovers between anaesthetists are frequent and it is not uncommon for more than one handover to occur during a single surgical procedure that lasts for 4 hours or more.

## Background

The Institute of Medicine (IOM) reported that health care in the USA was not as safe as it should be and estimated that as many as 44,000 people died each year of preventable errors (Kohn *et al*. 2000). The IOM also confirmed that certain areas of the hospital, including the operating room, intensive care unit and emergency room, are places where serious injuries are most likely to occur (Kohn *et al*. 2000). Not only is patient care compromised by communication errors, but healthcare costs increase as well. According to recent research, 12 billion dollars is wasted in the United States each year as a result of poor communication by healthcare providers (Agarwal & Sands 2008). Errors in communication negatively affect the well‐being of individual patients and of society by increasing the cost of health care. Adverse events such as wrong site surgery, prolonged length of stay and patient death are associated with communication errors between healthcare providers and increase the cost of health care (Kohn *et al*. 2000, Kluger & Bullock 2002, Agarwal & Sands 2008).

Communication errors between healthcare providers are likely to occur during handovers of care; in fact, communication failures during handovers of care are the most common factor contributing to the occurrence of adverse events in hospitalized patients (Bates 2003). A greater number of handovers leads to an increased likelihood of information being lost or distorted. The operating room, where handovers between providers commonly occur, is a uniquely complex environment with frequent distractions, interruptions and high noise levels. This environmental complexity may also contribute to lost information or inaccurate transfer of information during handovers in care (White 2004). Therefore, both the frequency of handovers and the complexity of the environment where they take place increase the risk of error during CRNA to CRNA handovers of care.

The Relationship‐Based Care (RBC) Model, developed by Koloroutis (2004), was the lens through which nurse perception of handovers was viewed in this study. In this model, the nurse knows his/her patient through relationships. The relationship between the nurse and patient and that between the nurse and colleagues are critical for truly knowing the patient. In this framework the structure of nurse work practices, such as handovers, is considered an essential element needed for high‐quality patient‐centred care (Koloroutis 2004). For CRNAs, truly knowing the patient and ensuring patient safety depend on timely, accurate and thorough transfer of information as CRNAs hand over the care of their patients to other CRNAs in the operating room setting. Little is published about such transfer of care between nurse anaesthetists in the operating room setting. There have, however, been several studies detailing the deficiencies of handover communications between anaesthesiologists and recovery room nurses. For example, in a recent study by Smith *et al*. (2008), observations of 45 handovers between anaesthetists and recovery room nurses were found to be brief, pressured, tension filled and prone to distractions. These authors observed a great deal of variability in the process of handover and omissions of important information were noted (Smith *et al*. 2008). It stands to reason that intraoperative handovers between CRNAs, in a similarly high‐pressured, tension‐filled and complex environment, would be equally prone to error. Therefore, this study examined the perceptions and experiences of CRNAs regarding handovers of care.

In studies focusing on intraoperative communications involving multidisciplinary providers, many errors and information omissions have been observed by researchers. Lingard *et al*. (2004) investigated the communication processes among providers in the operating room with trained observers witnessing 90 hours of surgery including 48 surgical procedures. The research team observed varied provider types including anaesthesiologists, surgeons, residents and nurses and documented communication exchanges in the operating room. During the 90 hours of observation, a total of 421 relevant communication events occurred and of these 129 were categorized as communication failures.

In a second observational study exploring multidisciplinary communication in the operating room by Halverson *et al*. (2010), researchers also found provider to provider communication to be highly error prone. In this investigation, a combination of field notes and a checklist was used to record communication events and errors involving inefficiency, delays, tension and procedures were commonly found (Halverson *et al*. 2010).

A retrospective review of malpractice claims reported communication breakdowns between providers during surgery as a common threat to patient safety and 43% of these breakdowns occurred during handovers in care (Greenburg *et al*. 2007). The majority of these handovers occur within professional groups rather than between professional groups. The culture of the provider group and the specifics of the situation are the underpinnings of any process of communication (Johnson & Arora 2009). Therefore, the way information is communicated during handovers can vary greatly across disciplines.

Some experts, recognizing that handovers are specific to the context where they occur, have studied interdisciplinary handovers, including those occurring between emergency room physicians (Ye *et al*. 2007), surgical residents (Borowitz *et al*. 2008), intensive care physicians (Pickering *et al*. 2009) and ward nurses (Meisner *et al*. 2007). There have been both similarities and differences found in these handovers. Physicians and nurses in various specialties perceive that inaccuracies and omissions in handover communications affect the safe continuity of patient care (Ye *et al*. 2007, Borowitz *et al*. 2008, Pickering *et al*. 2009). It is also apparent that handovers occur frequently, thereby increasing the potential for information loss or inaccuracy. Specific problems related to the specialty areas such as a lack of confidentiality during handovers between emergency room providers (Currie 2002), lack of face‐to‐face handovers among residents (Arora *et al*. 2005) and multi‐purpose ‘cheat sheets’ used by nurses for shift report (Hardey *et al*. 2000) have been identified. There are very few studies exploring handovers between nurse anaesthetists working in the operating room setting. This gap is surprising considering that nurse anaesthetists' handovers of care are frequent, highly complex and occur in the high‐risk area of the operating room.

Given the frequency of handovers and the known risk of ineffective handover communication, there is an urgent need to explore intraoperative handovers between nurse anaesthetists and the perceived threats to effective communication and patient safety in these handovers. This exploratory study has potential to guide future research aimed at development of interventions to improve handovers between healthcare providers, improve patient safety and quality of care and lower the cost of care.

## The study

### Aim

The aim of this study was to explore the perceptions and experiences of nurse anaesthetists regarding the process of intraoperative CRNA to CRNA handovers of care, including perceptions of facilitators and barriers to effective handovers.

### Design

This qualitative study was designed to use focus groups with CNRAs who have experience with handovers of patients undergoing general anaesthesia. There is no published research on CRNA to CRNA handovers in care; thus, this topic was explored qualitatively to hear the ‘voices’ of the providers who are involved with handovers on a day‐to‐day basis. The authors' philosophic bias is subjectivist in that personal context and situational context are thought to be irrevocably intertwined in reality; therefore, exploring handovers from the perspective of a particular population (CRNAs) and in a certain context (the operating room) is expected to increase knowledge of this process.

In focus groups, multiple participants are interviewed together and this type of interview has several distinct advantages (Hesse‐Biber 2011). Focus groups allow the researcher to inductively determine what the key ideas, issues and concerns are from multiple participants at once (Hsiu‐Fang 2005). The group affect in the focus group interview serves two important functions. Firstly, participants' words are affected by the attitude of the group, their views are reflected through the eyes of the other participants. Secondly, information is elicited not simply by the researcher, but by all group members, including the participants. Not only are more ideas produced, but the group interaction may empower participants to express their views (Hsiu‐Fang 2005).

### Sample

The participants of the focus groups were recruited from among persons who were former students from a nurse anaesthesia educational programme in northeastern United States and/or were preceptors involved with this same programme. All CRNAs who graduated from the programme between 2002–2012 and who were known to be employed in the greater Boston area, along with CRNAs who routinely mentor graduate students from this programme (multiple hospitals in the greater Boston area) were invited to participate in a focus group. Written email invitations were sent out to 20 CRNAs. Later, reminder telephone calls were made to ensure a high turnout at the focus groups. Ten CRNAs were unable to participate due to personal or scheduling difficulty. Thus, the purposive sample (Patton 2002) included 10 CRNAs.

### Data collection

The two focus groups took place between March 2012–October 2013 at a college located in the northeastern USA. There were five participants in each focus group, each focus group met once and Table 1 provides a detailed description of the sample characteristics.

**Table 1:** Description of participants.

	Focus group 1					Focus group 2				
	#1	#2	#3	#4	#5	#6	#7	#8	#9	#10
Characteristics of participants										
Graduate of this Programme (Y or N)	N	N	Y	N	N	Y	Y	Y	Y	Y
Gender (M or F)	F	F	F	M	F	M	F	F	F	F
Years of experience as CRNA^a^	H	H	I	H	H	L	L	I	L	L
Full‐Time (Y or N)	Y	Y	Y	Y	Y	Y	Y	Y	Y	Y
Tertiary (T) vs. Community (C) practice	C	C	T	C	T	T	C	T	C	T

a L = less experience (<3 years of practice as a CRNA), I = intermediate experience (3–5 years of practice as a CRNA); H = higher level of experience (>5 years of practice as a CRNA).

#### Facilitation of the interviews

DT, who has been educated in qualitative research at the doctoral level, facilitated the focus groups and an assistant (SE), with similar expertise in qualitative research, audio‐visually recorded the session and took field notes. Neither DT nor SE had any authoritative position over the participants as they were not current students or employees of the college.

The groups were begun with an introduction to the purpose of the study and instructions regarding confidentiality, mutual respect, turn‐taking in speaking and the time frame for the interview. An interview guide was used (Appendix A) and four open‐ended questions were developed on the basis of clinical experience. The questions were as follows:

1. What is your (the CRNA participant) experience of handovers in care from CRNA to CRNA intraoperatively?
2. What do you consider an effective handover?
3. What do you consider an ineffective handover?
4. What are the barriers and facilitators of effective handovers between nurse anaesthetists in the operating room?

The groups ended with a debriefing evaluation of the session. A low level of facilitator involvement was maintained deliberately to determine how the participants perceived handovers through the group conversations.

The focus groups were audio‐visually recorded using transcription conventions that indicated basic conversational turn‐taking. The recordings were transcribed by DT and the accuracy of the transcription was checked by SE and DT. Both focus groups lasted for 90 minutes, which is a typical time period taken to cover a topic to the satisfaction of the participants (Stewart *et al*. 2007).

### Ethical considerations

This study was approved by the Institutional Review Board of a major education institution and was conducted in accordance with the approved protocol. Prior to the start of the interview, consent was given, questions were answered and participants were informed that if they chose not to participate in the study at any point they could withdraw their consent without prejudice. Data were handled confidentially and the data extracts presented in the ensuing results section were de‐identified to protect the identity of the participants.

### Data analysis

Theme‐based content analysis was used, with a view towards the influences of Relationship‐Based Care between and among nurse anaesthetists and their patients. The data were transcribed word for word from the audio‐visual tapes by the primary investigator and the tapes were then re‐checked for accuracy. Computer assistance was used with HyperTranscribe software, which allowed the investigator to loop back the audio and visual data, listen intently to the participants' words and record non‐verbal behaviour. Content analysis was used to interpret meaning from the context of the text of the interviews. The interview was analysed to summarize the informational content of the data and the perceived effect of the participants' relationships with their patients and colleagues during handovers of care.

**Figure 1:**
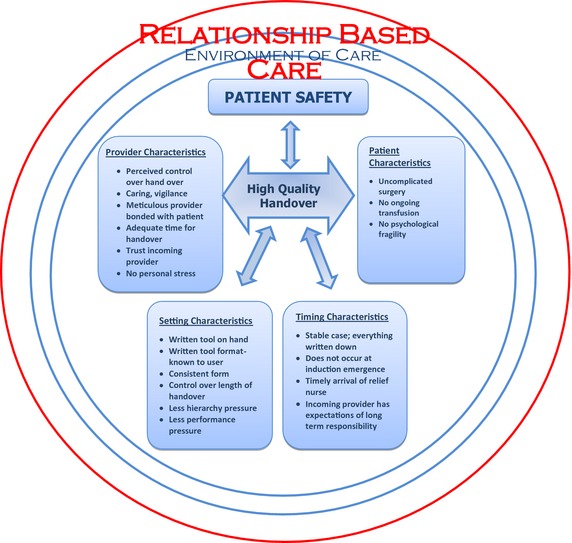
Model of Relationship‐Based Care, patient safety and high‐quality handovers of care; derived from interviews with nurse anaesthetists.

First, line by line coding was undertaken so as to remain as close as possible to the data. Secondly, memoing and focused coding were used to aid in linkage of analysis and interpretation and allow ideas to emerge. In the process of writing memos, the codes were raised to the level of a category and then four major themes were derived inductively from the data. The derived themes (Figure 1) were as follows: Challenges in the environment of care, individual provider characteristics, timing of the handover and individual patient characteristics have an impact on the quality of handover and patient safety. These four themes concerned the participants' descriptions of both beneficial and harmful influences that affect handovers between nurse anaesthetists.

The characteristics of each of the four themes were explored and described through systematic comparisons of the thematic content and the four themes were linked to exemplary data extracts. Lastly, the original audio‐visual recordings were re‐examined with the transcriptions to determine whether the data extracts and the four themes represented a balanced and thorough interpretation across the two interviews. The participants provided feedback on the analysis and conclusions of the study to ensure transparency in analysis and enhance the rigour of the study.

## Results

### Characteristics of the setting are a threat to handover quality

Participants perceived that the setting may either support or impede the process of handovers by CRNAs. Some important positive factors that lend themselves to improved handovers included: consistent, familiar, thorough, well‐designed pre‐anaesthesia evaluation forms. All of the participants used the pre‐anaesthesia form as a visual cue to provide information about the patients' history and physical status during every handover. The importance of this visual cue was pointed out clearly by Participant 1, who said that that if the provider is not familiar with the form, the organization of the handover was affected. In her own words: ‘the pre‐anesthesia evaluation form organizes your thoughts when you are giving report…if it is unfamiliar then your (handover) report is not as organized'. Accessibility of the pre‐anaesthesia assessment form at the time of the actual handover was thought to be a challenge as noted by Participant 7 who said: ‘Many different providers are using the same pre‐operative assessment form at the same time, they don't like you to take that paper off the chart, we can copy it, but if you don't have a chance then we don't have the information when we're giving the handover'. At times the pre‐anaesthesia assessment form is present, but is not complete, which was thought to cause problems during CRNA handovers, as articulated by Participant 1: ‘I can't tell you how many charts I've seen where people haven't filled out the airway assessment appropriately…that's one of the things you want to cover…airway…especially if you're going to be waking someone up…you really need to know'.

Challenges in the care setting were also thought to occur when the forms were poorly designed, as revealed by Participant 3: ‘Some pre‐anesthesia assessment forms are poorly designed and I feel that something is going to happen…these forms can have four pages of assessment and I look at it as someone is going to miss something on that 4 or 5 page pre‐op…Something is going to happen’. Extant literature demonstrates that incomplete notes used by healthcare providers during handovers contribute to handover errors (Arora *et al*. 2005), but the specific challenge with the forms used as a visual cue by CRNAs during handovers was a new finding. Participants unanimously supported standardization of the handover process, as does the Joint Commission (Joint Commission on Accreditation of Healthcare Organizations 2007), but participants spoke of unique challenges to implementation of a standardized handover process. Participant 4 remarked ‘we used to have this sheet to give report (posted handover guidelines) but no one ever took the time to go over that sheet and you know…it was not utilized. I think there needs to be buy‐in regarding standardizing the process, but I think it would be a great thing’.

The time allotted for handovers is known to be important (Joint Commission on Accreditation of Healthcare Organizations 2007), but specifics of this for CRNA handovers also brought forth the issue of hierarchical pressures. *Participant 1* clearly expresses this in the following statement ‘it just happened to me yesterday. I went to give report to a senior anesthetist and I get the reaction ‘I know how to read GO, GO, GO’ I know that the incoming provider can read but he is not familiar with this patient and the IV, you know, because of the arm position, could malfunction’.

### Individual provider characteristics have an impact on handover quality

The second theme that arose was that individual provider characteristics affect handover accuracy and completeness. Participants agreed that the CRNAs who are most caring, vigilant and meticulous tend to give more accurate and complete handovers. Participant 2 said: ‘a caring, vigilant anesthetist, you know, checks the ID, checks the allergies…because they really care about the person, so they care to do the right thing in the handover’. When provider feels they have a bonded relationship with the patient they are less likely to hand over the care of the patient to a second provider. Participant 6 expressed this in the following way: ‘If you communicate with the patient, then you put the patient to sleep, I think you as the initial provider have a better sense of the patient than someone who comes in and takes over…because once you've bonded with the patient…it seems.. I always feel like if I start the case I finish it’

Lack of trust in the incoming provider was thought to have a negative impact on handovers between CRNAs. Respondents thought that some anaesthetists are better listeners than others and they feel more trust that the handover was effective when reporting to those anaesthetists. Participant 10 remarked: ‘some people I give report to and I walk away and wonder if she was even listening…I feel like that handover wasn't good’

Stressful personal circumstances by stressful personal circumstances, such as the relief anaesthetist arriving later than expected when the primary anaesthetist had an appointment after work. This was expressed concisely in the following statement by Participant 4: ‘communication is so important and it gets forgotten because of stress…I think it (handover) would probably be better on a day where I didn't have any stress or a place I had to get to, as opposed to having so much on my mind’. Fatigue was also thought to negatively affect handovers between CRNAs. Fatigue and stress have been previously linked to medical errors (Wachter & Shojania 2004), but fatigue effecting handovers is a new finding. Some participants perceived gender to be a factor in handover effectiveness, whereas other participants felt that gender was unimportant and all participants felt that experience level did not have an impact on handover effectiveness.

### Challenges in the timing of handover are a threat to handover quality

The third major theme uncovered was that the timing of handovers was thought to affect the quality of the handover. Participants expressed concern that reports were much abbreviated when it was thought that the relief would last for a short period of time, such as for a coffee break, which typically lasts for 15 minutes. This is voiced clearly by Participant 3 as follows: ‘for a 15 minute break you're given the bare minimum (report) and you don't know what's going to happen when you're in there.’ In addition, handovers that occur shortly after induction or close to emergence from anaesthesia were thought to carry a higher risk. Participant 7 noted: ‘frequently right after induction tasks are not completed…(I) fear things will go wrong…IV in the wrong arm, antibiotic timing mistakes, medication errors such as (inadvertent) use of long acting muscle relaxants’. These challenges have not been reported; hence the timing of handover threat to patient safety appears to be unique to nurse anaesthesia practice.

### Individual patient characteristics have an impact on handover quality

The fourth major theme that arose was that patient characteristics are perceived to affect the quality of handovers. Handovers were thought to be likely to be effective when patients were in good general health and experiencing less complex surgeries. Participants agreed that patients with high acuity, difficult intraoperative course, undergoing transfusion, or who experienced psychological distress pre‐operatively would be at higher risk of handover error and that they would be more likely to stay with that patient and refuse to hand over the care. Participant 9 articulated this in the following way: ‘if the patient has lost liters of blood, you've given blood products…you have an arterial line in…you're questioning if you are going to leave the patient intubated…that handover would be difficult…I would try to stay’.

Participants also revealed that if the patient were highly anxious, it would be difficult to effectively hand over the patient because the incoming provider would not know the patient as well. Participant 10 portrayed this as follows: ‘for instance, yesterday I had a patient with head injury…he was very paranoid, so you know there was a lot of anxiety going on…and I'm afraid that would get lost in the translation (during handover)’. The specific factors of high blood loss, blood transfusion in progress and psychological fragility in patients represent new findings.

## Discussion

Participants described many factors in the care setting, timing of handovers and both provider and patient characteristics that had an impact on their ability to safely hand over the care of patients in the operating room. These findings are consistent with previous research on both nurse and physician perceptions and experiences with handovers (Meisner *et al*. 2007, Cleland *et al*. 2009 Nagpal *et al*. 2010).

Specific setting characteristics, including the accessibility of information pertinent to the handover, a consistent pre‐anaesthesia evaluation form used as a visual cue during the handover and control over the length of the handover, were identified as improving the accuracy and effectiveness of intraoperative handovers of care between nurse anaesthetists. This finding is in agreement with a recent report, which concludes that setting features including lack of time, unfamiliarity with the handover process and lack of standardized process interfere with safe, effective handovers of care between nurses on hospital wards (Blouin 2011).

Provider characteristics such as degree of caring, vigilance and trust in the incoming provider were thought to affect the quality of the handover. This is a new finding, but previous research involving handovers between physicians found that professional attitude is critically important to safe handovers of care (Cleland *et al*. 2009). These authors explored physician perception of handovers via focus group interviews and found that provider characteristics of attentive listening, good team work and professionalism were perceived as the most important characteristics. Although provider characteristics were thought to be important to handover in both this study and Cleland study, the specific characteristics of the provider that enhanced the handover differed. There was concurrence in the research regarding certain elements of the setting, for example, participants in both the (Cleland *et al*. 2009) study and in this research expressed their perceptions that careful listening, lack of interruption and a calm unrushed setting were necessary for effective handovers.

These results differ from previous research by (Meisner *et al*. 2007) in that the timing of the handover was perceived to affect the handovers in this study setting. Meisner investigated nurse perception of handovers in Europe and found that the timing of the handover, whether it occurred at night or during the daytime, was not considered a factor. The population of participants differed greatly between studies as the data collected in the Meisner study included all nurses working in hospitals, whereas this research is specific to handovers of care between nurse anaesthetists in the operating room.

Finally, these participants described patient characteristics as being important to handover effectiveness. This is similar to previous research by Perry (2004) and Simpson (2005), who found that incoming and outgoing providers may view complex care differently and this may have an impact on handover effectiveness. Additional issues were voiced by these participants, including an increased perceived risk when handing over patients whose condition is complex either physically or psychologically and, therefore, may not be as well understood by the oncoming nurse anaesthetist.

### Limitations

Certain situational conditions affected how the data set was produced and therefore influenced the findings. Study participants were recruited through a list of former students or current preceptors of one nurse anaesthesia programme. Participants’ experiences may have been framed by the involvement with the programme. Although participants were employed at varied types of hospitals (Table 1), all were connected to the nurse anaesthesia programme, were located in northeastern U.S. and worked within an anaesthesia care team with anaesthesiologists and nurse anaesthetists. This homogeneity of participants limits the generalizability of the findings.

Second, the group composition differed between the two groups with the second group having less experience on average than the first group (Table 1). Additional data are needed and follow‐up interviews would provide more depth to the understanding of handovers in this setting.

#### Implications for practice

Despite the stated limitations, this research identified four themes that could strengthen or undermine the quality and safety of handovers of care between Certified Registered Nurse Anaesthetists (CRNAs). High‐quality handovers are associated with the CRNA having sufficient time for the handover report, having control over the handover and having trust in the incoming provider. Therefore inadequate time, control, or insufficient knowledge of the incoming provider could increase the risk when care is handed over between CRNAs. Handovers are thought to be higher risk when they occur during complicated surgeries and during blood product transfusion; therefore, handovers should be limited during these time periods. Hierarchical pressure and performance pressure lead to an increased risk of handover quality and should be balanced by the overriding concern for high‐quality handovers, particularly in the high‐risk area of the operating room. Likewise, the specific timing of the handover should be considered and, if possible, should be limited to stable periods of the operation rather than during induction or emergence from anaesthesia.

## Conclusion

Handovers may be problematic in many disciplines and there are common threats to the accuracy of handover communications. Nurse anaesthetists perceive unique threats to handover effectiveness in the operating room. Deepening our knowledge of these specific threats to high‐quality handover between nurse anaesthetists in the operating room will enable the design of interventions and policies to reduce these threats and improve patient safety.

This study is a first step in identifying what factors promulgate effective handovers and what barriers exist to effective handovers. Further research, such as a survey of CRNA perception of handovers, would be needed before generalizing this information. If shown to be generalizable to a broader population of nurse anaesthetists, this knowledge has far‐reaching potential import for future education, practice and policy development.

## Conflict of interest

No conflict of interest has been declared by the authors.

## Author contributions

All authors have agreed on the final version and meet at least one of the following criteria [recommended by the ICMJE (http://www.icmje.org/ethical_1author.html)]:

- substantial contributions to conception and design, acquisition of data, or analysis and interpretation of data;
- drafting the article or revising it critically for important intellectual content.

## Appendix A Interview guide

Does anyone have questions or concerns about this consent or about the study? It is important that you know that you are free to remove yourself from this study at any time without prejudice. We are very interested to know your experience and perceptions of handovers between CRNAs and I am here as the moderator. I hope to hear all of your voices, so it would be best if only one person spoke at a time and if all of you tell me about your perceptions and experiences in this area. This group will be audio‐visually recorded. I will transcribe the tape and will ensure that this entire interview is kept completely confidential. Does anyone have a question? If not, let's get started

1. What is your experience of handovers in care from CRNA to CRNA intraoperatively?
  Prompt – can you describe a usual handover in care?
2. What do you consider an effective handover?
  Prompt – Can you describe an experience of an effective handover?
  Prompt – Are there facilitators to this, meaning things that have an impact on a handover so that it is more effective?
3. What do you consider an ineffective handover?
4. What do you perceive as the barriers to effective handovers between nurse anaesthetists in the operating room?
